# Effects of ginger (*Zingiber officinale*) on gingival fibroblasts: An in vitro study

**DOI:** 10.1002/cre2.575

**Published:** 2022-04-05

**Authors:** Nouf Al‐Shibani, Reem Al‐Kattan, Lamees Alssum, Eman Allam

**Affiliations:** ^1^ Department of Periodontics and Community Dentistry, College of Dentistry King Saud University Riyadh Saudi Arabia; ^2^ Oral and Dental Research Division National Research Centre Cairo Egypt; ^3^ European University College Dubai United Arab Emirates

**Keywords:** human gingival fibroblasts, interleukins, matrix metalloproteinases, *Zingiber officinale*

## Abstract

**Objectives:**

Ginger, the powdered rhizome of the herb *Zingiber officinale*, is commonly used as a traditional medicine in many areas around the world. Anti‐inflammatory actions of its extract have been previously reported. The aim of this study was to investigate the effect of ginger extract on matrix metalloproteinase (MMP) and interleukin (IL) expression from human gingival fibroblasts (HGFs) in vitro.

**Material and Methods:**

HGFs were obtained from subcultures of biopsies from clinically healthy gingival tissues of 10 patients. Ginger extract was prepared from commercial powder of rhizome of *Z. officinale* (GZO) and its effect on cell viability was assessed using the 3‐[4,5‐dimethylthiazol‐2‐yl]‐2,5 diphenyl tetrazolium bromide cytotoxicity assay. Cells were then incubated and treated (except for the control samples) with either GZO, lipopolysaccharides (LPS), and GZO before or after LPS stimulation. Culture supernatants of all five samples were collected for the Milliplex analysis to measure MMP‐1, MMP‐2, MMP‐8, MMP‐9, IL‐1β, and IL‐8. One‐way analysis of variance and Duncan multiple range tests were used to compare the mean values of all groups.

**Results:**

The gingerextract showed minimal cytotoxicity to HGFs even with the maximum tested concentration. Compared to the control group, GZO treatment alone caused little or no effect on the levels of expression of MMP‐1, MMP‐2, MMP‐8, MMP‐9, IL‐1β, and IL‐8. While GZO treatment after LPS stimulation significantly reduced the expression of MMP‐1, MMP‐2, MMP‐8, MMP‐9, and IL‐8 when compared to LPS alone. Comparing the control to LPS stimulation after GZO treatment, significant differences were detected for all tested MMPs and cytokines.

**Conclusions:**

These findings suggest a potential role for ginger extract in inhibiting MMP and IL HGFs' expression in inflamed gingival tissues.

## 1 INTRODUCTION

Periodontal diseases are infectious diseases characterized by periodontal attachment loss and bone destruction. They are considered one of the most common chronic debilitating diseases affecting a large number of people, causing considerable pain and discomfort, and are costly to control and treat. Their impact is, therefore, significant to both the individual and the wider community. The main etiological infectious agent involved in the pathogenesis of periodontitis is the Gram‐negative bacteria, such as *Porphyromonas gingivalis* (Pg), *Aggregatibacter actinomycetemcomitans*, *Prevotella intermedia*, and *Tannerella forsythia*.  These microbial agents trigger a complex host immune‐inflammatory reaction leading to the characteristic tissue destruction response (Kinane, 2001; Lovegrove, 2004; Popova et al., 2013; R. Saini et al., 2009).

Human gingival fibroblasts (HGFs) are the main cell type residing in gingival mesenchymal tissues and are responsible for the integrity of the extracellular matrix. They produce the essential gingival connective tissue proteins and glycoproteins as well as most of the immunoregulatory cytokines and chemical mediators including matrix metalloproteinases (MMPs). Their role is deemed to be crucial in periodontal disease pathogenesis and tissue destruction. Regulation of HGFs cellular reaction has been suggested as an essential pathway in preventing and controlling the progression of periodontal tissues' pathological alterations as well as healing processes (Dahan et al., 2001; Page, 1991).

Ginger (botanically known as *Zingiber officinale*) is a herbaceous plant that has been, for centuries, extensively used in many regions around the world as a spice. While India is considered the biggest producer of ginger in the world, the plant is also grown extensively in other countries, such as China, Nepal, the United States, Bangladesh, Taiwan, and Jamaica among other parts of the world. *Z. officinale* is also commonly used as a medicinal plant in Chinese, Indian Ayurvedic, and Muslim's Tibb‐Unani herbal medicines for the treatment of many diseases, including asthma, stroke, digestive problems, rheumatoid arthritis, nervous diseases, and diabetes (Bhatt et al., 2013; Khalil et al., 2011; Moghaddasi & Kashani, 2012; Santo & Sankari, 2017). *Z. officinale* has previously been shown to possess an anti‐inflammatory effect in an in‐vitro periodontal disease model as well as in an in vitro *Candida albicans* model (Eslami et al., 2015; Koontongkaew et al., 2013). The purpose of this study was to assess the effect of *Z. officinale* on the HGFs' MMPs (MMP‐1, MMP‐2, MMP‐8, and MMP‐9) and inflammatory cytokines (interleukin [IL]‐1β and IL‐8) expression as an attempt to explore potential scientific bases for the claims of its anti‐inflammatory role and the benefits of its use in several commercially available ginger‐containing mouthwash. Effects of ginger extract were tested in vitro on cultured HGFs, in the presence or absence of lipopolysaccharide (LPS).

## 2 MATERIALS AND METHODS

### 2.1 Cell collection and culture

HGFs were obtained from subcultures of gingival biopsies from clinically healthy gingival tissues of 10 different patients (14–34 years old) undergoing periodontal surgeries at the College of Dentistry, King Saud University Hospital, KSA. Tissue collection was done by the principal investigator (N. A.‐S). Tissues were transported to the laboratory in phosphate‐buffered saline (PBS) solution, washed with 70% ethanol, and rinsed in PBS several times to remove the ethanol. Tissues were then cut into small fragments of approximately 1 mm^3^ and placed in cell culture dishes, air dried, and incubated for 5–7 days at 37°C and 5% CO_2_ in low‐glucose (100 mg/L) Dulbecco's modified Eagles medium supplemented with 15% fetal bovine serum (Hyclone, Logan, Utah, United States), Two hundred millimoles of l‐glutamine, 100 U/ml penicillin and 50 μg/ml gentamycin that was changed every 2 days. Explants were subcultured out of the cells that grew out and cells at passages 4–8 were used in the experiments (Al‐Shibani & Windsor, 2008; P. Saini et al., 2012). The study was approved by the Institutional Review Board of King Saud University (project number E‐20‐4713). Subjects who participated in this study were provided with a detailed explanation of the experiment and purpose of the research and signed informed consent for participation.

### 2.2 Preparation of ginger solution

The ginger solution was prepared from commercial powder of rhizome of the ginger plant (*Z. officinale*: GZO) (DRUERA; Dru Era Pty Ltd., Wilmington, DE, USA) The extraction was done by dissolving 40 g of powder in 200 ml of distilled deionized water and incubated at room temperature for 24 h with shaking at 120 rpm. After extraction, the solids were removed by centrifugation at 3000*g* at 25°C for 10 min and filtered with a 0.22 μm membrane filter, and then stored at −80°C.

### 2.3 Cellular cytotoxicity assay

Cellular cytotoxicity was assessed using the MTT (3‐[4,5‐dimethylthiazol‐2‐yl]‐2,5‐diphenyl tetrazolium bromide)‐based cytotoxicity assay. HGFs were seeded in 96‐well plates at a density of 2 × 10^5^ cells/well in a 100 µl optimized medium. The total number of cells was determined by the trypan blue exclusion test (0.4%) using a cell counter. Cells were allowed to settle for 24 h before treatment with individual serial concentrations of GZO (6.25, 12.5, 25, 50, and 100 µg/ml) except for the control. Treated cells were allowed to grow further for 48 h. At the end of the incubation period and concentration point, 20 µl of Cell Titer 96® Aqueous One Solution Cell Proliferation Assay (Promega, Madison, Wisconsin, United States) was added at 37°C at the final concentration of 5 mg/ml. The 96‐well plate was kept in the dark for 2 h. The optical density (OD) of each treatment was measured at absorbance 490 nm using a 96‐well plate reader (Molecular Devices—SPECTRA max—PLUS384) and was performed in four replicates. Values of ODs were normalized according to the control (untreated cells). Therefore, cell viability values of untreated cells were 100% while values of treated cells had values below 100%. The GZO concentration lethal to 50% of cells was calculated from appropriate dose‐response curves.

### 2.4 Treatment of cells

HGFs (150,000 cells/ml), using a haemacytometer (Hawksley, UK), were seeded in 3 ml plates and incubated in a complete medium at 37°C overnight. Cells incubated in a culture medium without LPS or GZO extracts were used as a control (Sample 1). Cells treated with 50 µg/ml of GZO and incubated for 24 h were considered as Sample 2. Sample 3 consisted of cells stimulated with 5 μg/ml Pg LPS (InvivoGen) for another 24 h. Sample 4 consisted of cells stimulated with LPS 5 µg/ml for 24 h before being exposed to GZO (50 µg/ml) while Sample 5 included cells pretreated with GZO 50 µg/ml for 24 h before being exposed to LPS (5 µg/ml). Culture supernatants of all five samples were collected for the Milliplex analysis.

### 2.5 Milliplex analysis

Immunoassay analysis was performed by using xMAP technology (Luminex 200; Luminex, Austin, TX, USA) to measure MMP‐1, MMP‐2, MMP‐8, MMP‐9, IL‐1β, and IL‐8. The Milliplex MAP multiplex assay Human MMP Panel 2 Magnetic Bead HMMP2MAG‐55K and HSP2MAG‐63K (Millipore, Billerica, MA, USA) kits were used. Analysis was conducted in 96‐well microplate format according to the manufacturer's recommendations. Concentrations of MMPs were determined on the Bio‐Plex Protein Array System (Bio‐Rad, Hercules, CA, USA). Briefly, each of the bead solutions was transferred into a mixing vial and adjusted to a volume of 3 ml with bead diluents. Internal controls and standards were included in every assay. Following the addition of sample supernatants and beads, the resulting mixture was incubated overnight at 4°C. The plate was scanned using a Bio‐Plex array reader with the Luminex 200 software system.

### 2.6 Statistical analysis

Data were analyzed using SPSS 21.0 version (IBM Inc., Chicago, IL, USA) statistical software. One‐way analysis of variance test and Duncan's multiple range tests were used to compare the mean values of the study groups. A *p* < .05% and 95% confidence intervals were used to report the statistical significance and precision of mean values.

## 3 RESULTS

**Figure 1:**
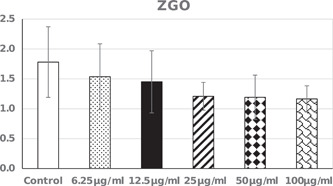
Cell viability assay values (mean and standard deviation). Comparison between groups done using one‐way analysis of variance test with Dunnett test as multiple comparisons to compare each group with the control. GZO, *Zingiber officinale*

Potential cytotoxicity of GZO was measured by MTT cell viability assay. Five different concentrations of GZO were tested (6.25, 12.5, 25, 50, and 100 μg/ml) and testing was repeated three times. Results indicated limited cytotoxicity to HGFs since viability assay values showed nonsignificant differences compared to the control even with the maximum tested concentration (Figure 1). Cytotoxicity was not statistically significantly different from the control for 6.25 µg/ml (*p* = .934), 12.5 µg/ml (*p* = .822), 25 µg/ml (*p* = .407), 50 µg/ml (*p* = .383), and 100 µg/ml (*p* < .344).

**Figure 2:**
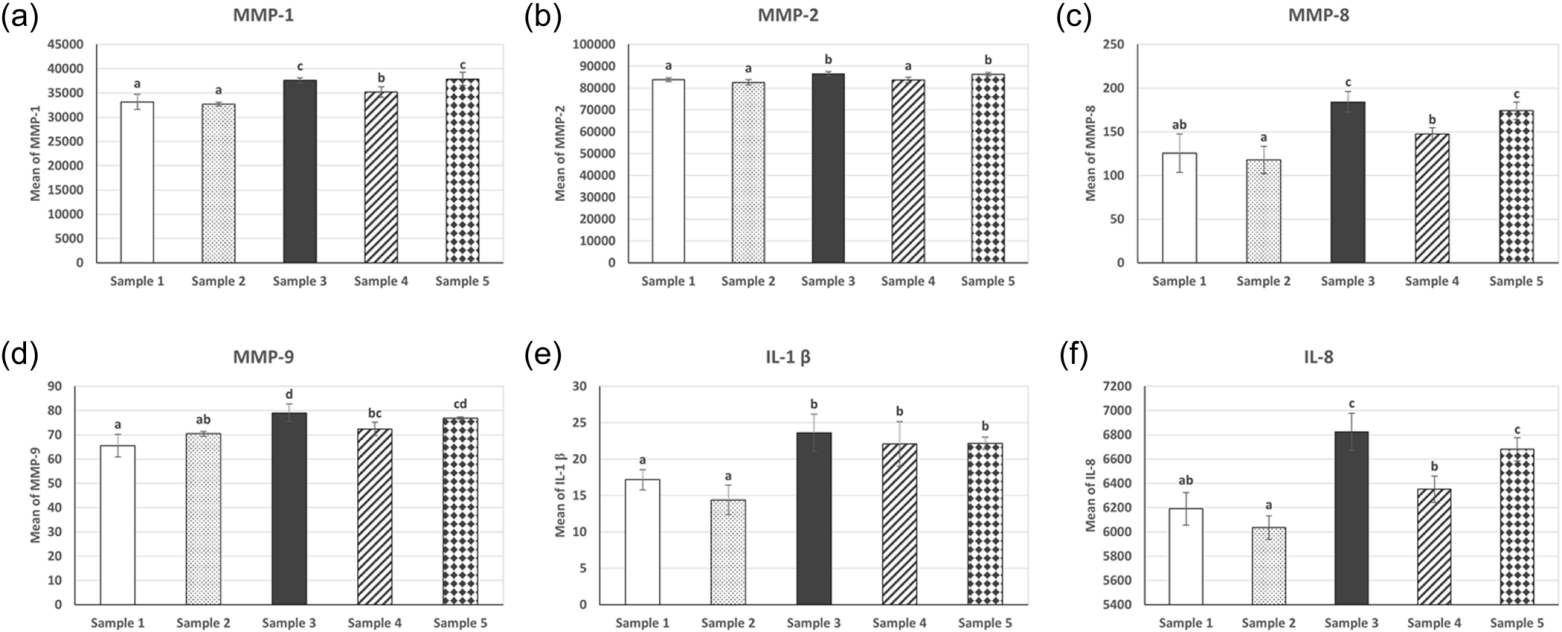
Mean and standard deviation of all groups for (a) MMP‐1, (b) MMP‐2, (c) MMP‐8, (d) MMP‐9, (e) IL‐1 β, and (f) IL‐8. The groups that have the same letter are not significantly different. IL, interleukin; MMP, matrix metalloproteinases

The effect of GZO on HGF expression of MMPs, IL‐1β, and IL‐8 levels was determined by a comparison between all groups including the control group, as shown in Table 1 and Figure 2. Compared to the control group, GZO treatment alone caused little or no effect on the levels of expression of MMP‐1, MMP‐2, MMP‐8, MMP‐9, IL‐1 β, and IL‐8; while GZO treatment either before or after LPS stimulation significantly increased MMP1, MMP8, MMP9, IL‐1β, and IL‐8 levels. As expected, LPS significantly increased all levels of tested MMPs and cytokines while GZO treatment after LPS stimulation significantly reduced the expression of MMP‐1, MMP‐2, MMP‐8, MMP‐9, and IL‐8 as compared to the LPS only. Significant differences were detected for all tested MMPs and cytokines when comparing control to LPS stimulation after GZO treatment (Sample 5).

**Table 1:** Comparing groups using one‐way analysis of variance test with Duncan's test as multiple comparisons

Parameter	Groups	Min.	Max.	Mean ± standard deviation	Percent change	*p* Value
MMP‐1	Control	32,109.00	34,955.00	33,136.00 ± 1579.68^a^	100.00	.001
	GZO	32,453.00	33,120.00	32,687.67 ± 374.87^a^	98.65	
	LPS	37,093.00	38,099.00	37,582.33 ± 503.56^c^	113.42	
	LPS and GZO	33,933.00	35,965.00	35,163.67 ± 1081.90^b^	106.12	
	GZO & LPS	36,854.00	39,444.00	37,877.33 ± 1377.84^c^	114.31	
MMP‐2	Control	83,342.00	84,815.00	83,863.00 ± 825.68^a^	100.00	.003
	GZO	81,264.00	83,340.00	82,611.00 ± 1167.86^a^	98.51	
	LPS	85,473.00	87,470.00	86,518.33 ± 1001.79^b^	103.17	
	LPS and GZO	82,324.00	84,733.00	83,661.33 ± 1226.28^a^	99.76	
	GZO and LPS	85,436.00	86,755.00	86,288.67 ± 739.51^b^	102.89	
MMP‐8	Control	104.43	148.45	125.44 ± 22.08^ab^	100.00	.001
	GZO	100.43	130.54	117.80 ± 15.58^a^	93.91	
	LPS	175.44	197.54	184.10 ± 11.80^c^	146.76	
	LPS and GZO	142.34	155.44	147.56 ± 6.94^b^	117.63	
	GZO and LPS	166.54	185.51	173.90 ± 10.17^c^	138.63	
MMP‐9	Control	60.43	69.70	65.52 ± 4.70^a^	100.00	.002
	GZO	69.43	71.32	70.43 ± 0.95^ab^	107.49	
	LPS	76.43	83.23	79.07 ± 3.65^d^	120.68	
	LPS and GZO	70.32	75.54	72.43 ± 2.75^bc^	110.55	
	GZO and LPS	76.43	77.34	76.85 ± 0.46^cd^	117.29	
IL‐1 β	Control	16.33	18.76	17.17 ± 1.38^a^	100.00	.001
	GZO	12.06	15.77	14.38 ± 2.02^a^	83.75	
	LPS	21.43	26.44	23.62 ± 2.56^b^	137.56	
	LPS and GZO	19.43	25.43	22.06 ± 3.07^b^	128.47	
	GZO and LPS	21.32	23.00	22.18 ± 0.84^b^	129.13	
IL‐8	Control	6047.00	6312.00	6190.67 ± 133.90^ab^	100.00	.001
	GZO	5933.20	6127.10	6034.87 ± 97.29^a^	97.48	
	LPS	6649.30	6932.20	6824.90 ± 153.32^c^	110.24	
	LPS and GZO	6283.90	6475.90	6351.03 ± 108.24^b^	102.59	
	GZO and LPS	6578.40	6766.40	6680.20 ± 94.97^c^	107.91	

*Note*: For each parameter, groups with the same letter are not significantly different.

Abbreviations: GZO, ginger *Zingiber officinale*; IL, interleukin; LPS, lipopolysaccharide; MMP, matrix metalloproteinases.

## 4 DISCUSSION

One of the key destructive pathways in periodontitis is the loss of connective tissue attachment as a result of collagen fiber degradation. The extracellular proteolytic system that is involved in periodontal tissue breakdown and is responsible for this collagen degradation includes mainly MMPs. IL‐1 and IL‐8 are also important inflammatory mediators produced as a part of the host response and significantly contribute to tissue destruction. Studies have indicated that their levels directly correlate with the severity of periodontal disease. Higher levels of inflammatory cytokines and metalloproteinases are significantly associated with the severity of the periodontal disease and the extent of tissue destruction (Gamonal et al., 2000; Silva et al., 2007; Yucel‐Lindberg & Båge, 2013).

Ginger is the rhizome of the plant *Z. officinale*, widely used as a spice, delicacy, and traditional medicine around the world. Its name stems from the genus or family (Zingiberaceae). Other well‐known members of this herbal family are turmeric, cardamom, and galangal. Preliminary research indicated that active compounds found in ginger may possess antimicrobial, antioxidant, anti‐inflammatory, and antinecrotic activities (Ali et al., 2008; Grzanna et al., 2005; Shahrajabian et al., 2019), which may explain its occasional use as an added ingredient in some of the commercially available mouthwashes as well as in some of the homemade mouthwash recipes. In this study, we investigated the effects of the ginger extract on MMPs and inflammatory cytokines expression from HGFs in vitro.

It is clear from the data of the current study that, compared to the control group, GZO treatment alone caused little or no effect on the levels of expression of MMPs, IL‐1, or IL‐8. Nevertheless, the data also indicated that when compared to the LPS‐stimulated HGFs, GZO treatment resulted in a significant reduction in the levels of expression of MMP‐1, MMP‐2, MMP‐8, MMP‐9, and IL‐8. Given the fact that the latter would more closely simulate the condition of periodontitis‐affected inflamed tissues, these findings may partially confirm the claims of using GZO as an effective botanical anti‐inflammatory compound in mouthwashes. We might also hypothesize that, based on these findings, while GZO may not have any potent effect on healthy gingival tissues, its effect may alleviate the inflammatory response and reduce the rate of progression of tissue degradation in periodontally affected tissues.

Several studies have evaluated ginger's effectiveness as an anti‐inflammatory agent. Previous in vitro studies suggested that ginger extracts block the formation of inflammatory compounds, such as prostaglandins and leukotrienes. In addition, animal data showed that in rat models of severe inflammatory arthritis, ginger oil effectively reduced inflammation and swelling (Flynn et al., 1986; Kiuchi et al., 1982; Sharma et al., 1994). Case series of patients with rheumatoid arthritis reported varying degrees of relief in pain and swelling and improved symptoms following supplemental ginger (Srivastava & Mustafa, 1989). In the current study, ginger extract resulted in a significant reduction in the LPS‐stimulated HGFs' expression of IL‐8.

IL‐8 is a regulatory cytokine that controls the immune response and inflammatory reaction of the gingival tissues in periodontitis. It is predominantly produced by polymorphonuclear leukocytes, HGFs, gingival crevicular epithelial cells, and periodontal ligament cells (PDL). Previous reports indicated that periodontal microbial infection enhances MMP‐8 gene and protein expression from HGFs and PDL cells and that the amount of MMP‐8 in the gingival crevicular fluid, gingival tissue, and PDL cells significantly increased in patients with chronic periodontitis (Escalona et al., 2016; Finoti et al., 2017).

Similar to our findings, Lantz et al. (2007), studying the effects of extracts from ginger rhizome on inflammatory mediator production using an in vitro test system, have shown that compounds found in ginger have anti‐inflammatory effects and are highly effective at inhibiting LPS‐induced production of PGE2. Koontongkaew et al. (2013) reported that the *Zingiber cassumunar* (another herbal plant from the same Zingiberaceae family) extract inhibited the expression of cyclooxygenase (COX)‐1, COX‐2, and MMP‐2 in HGFs challenged with LPS. Menon et al. (2020) compared the effectiveness of ibuprofen and dried ginger powder on pain and gingival inflammation following open flap debridement. They reported that, in 10 patients with chronic periodontitis, the differences in the pain scores and the degree of gingival inflammation assessed by the Modified Gingival Index were not statistically different and concluded that the effectiveness of the ginger powder for the management of pain and gingival inflammation following open flap debridement is comparable to that of ibuprofen.

Eslami et al. (2015) assessed the efficacy of ginger mouthwash as a therapeutic modality in the treatment of denture stomatitis as compared to the most commonly used antifungal mouthwash nystatin. In a cohort of 30 patients, they indicated that the efficacy of both treatments was similar, especially in improving inflammation and erythema after a 20‐day treatment period. They reported that patients were more satisfied with ginger mouthwash due to fewer side effects and it could possibly be recommended as an alternative to nystatin mouthwash in the treatment of denture stomatitis.

Targeting the MMPs has been traditionally suggested as an adjunctive therapeutic option in periodontal diseases since the progression of the disease is marked by increased collagen destruction, partly mediated by fibroblasts and their secretion of those factors. Our results showed that GZO significantly decreased MMP‐2 and MMP‐9, also referred to as gelatinases, as well as MMP‐1 and MMP‐8, the two main collagenases, expression in HGFs when compared with LPS‐stimulated HGFs. Therefore, we hypothesize that GZO active ingredients may alleviate periodontal proteolysis and ease inflammation in the periodontitis‐affected gingival tissues. These suggestions, however, remain to be further confirmed in pharmacokinetic and clinical studies.

In conclusion, this study has shown that ginger extract would potentially be an effective natural botanical additive that may help alleviate inflammation and tissue destruction characteristics of periodontal diseases. In addition, it is expected to be safe and possesses minimal side effects on the tissues, given its slight adverse effect on cellular viability as demonstrated by the results of the cytotoxicity assays.

## AUTHOR CONTRIBUTIONS

Nouf Alshibani, Reem Alkattan, Lamees Alssum, and Eman Allam contributed to the design of the study, performed the experiments, and participated in the analysis of the results as well as writing and revising the manuscript.

## CONFLICTS OF INTEREST

The authors declare no conflicts of interest.

## Data Availability

Data are available on request from the authors.
